# The influence of the University of Pennsylvania on UK dentistry 1880-1922

**DOI:** 10.1038/s41415-025-8332-0

**Published:** 2025-03-28

**Authors:** Chris Stephens

**Affiliations:** Emeritus Professor of Child Dental Health, University of Bristol, Bristol, UK

## Abstract

British attendance at the two early dental meetings held in Philadelphia in 1926 has recently been reported; however, the influence of the University of Pennsylvania (UPenn) on United Kingdom (UK) dentistry started much earlier. In March 1879, the first Doctor of Dental Surgery (DDS) degrees from UPenn were awarded, and during the next 40 years, well over 100 dentists travelled from the UK to obtain the DDS UPenn. Of these, 90 returned to practise in the UK. This paper considers the influence that these had on UK dentistry.

## Key points

- Describes the origins of the University of Pennsylvania Dental School.
- Throws light on the influence of the University of Pennsylvania Dental School on British dentistry in the early twentieth century.
- Identifies some of the origins of the emerging United Kingdom dental specialties of oral surgery and orthodontics.

## Introduction

Although the first Licence in Dental Surgery examination had been held by the Royal College of Surgeons of England (LDSRCS Eng) in March 1860, followed some 20 years later by the other United Kingdom (UK) surgical colleges,^1^ there was no compulsion for practising dentists to hold such a qualification at this time. However, the continuing pressure for dental reform culminated in the passing of the Dentists Act of 1878, which established the Dentists Register of the General Medical Council. Thereafter, no-one practising dentistry in the UK could describe themselves as a dentist, dental practitioner or any name, title or description that implied this unless they were so registered, but many unqualified people continued to practise by describing themselves in other terms.^2^ That situation did not change until the passing of the Dentists Act of 1921.

After the Register was introduced in 1878, in addition to the UK qualifications, those of two American universities were accepted for registration: the Doctor of Dental Medicine (DMD) (Harvard) and the Doctor of Dental Surgery (DDS) (Michigan). The first American to be so registered was Thomas William Hogue in April 1879, whose DMD Harvard had been achieved in 1870. The earliest UK-registered DDS Michigan was that of William Mitchell in 1885, whose degree had been obtained in 1878. By 1893, there were 23 UK dentists who had been registered in the UK on the basis of one of these two American qualifications.

According to Asbell, in 1885 and again in 1890, the Deans of the University of Pennsylvania (UPenn) Dental School had applied unsuccessfully to the Dental Board of the UK General Medical Council for recognition of the DDS UPenn as a registrable UK dental qualification.^3^ The reasons for this are unknown. However, by 1925, a single DDS UPenn graduate appeared in the UK Dentists Register's foreign list: that of Arthur Hitchcock Myers (1894-1957), whose DDS degree had been obtained in 1916.^4^ Maybe by this time, the DDS UPenn was accepted for registration purposes by the UK Dental Board. Before this time, those who had undertaken the DDS UPenn degree course invariably added ‘& Penna' or ‘Univ. Penna' after the name of their UK dental school to their entry in the UK Dental Directory. This was an independent publication compiled from the returns of an annual circular sent to every UK graduate and Licentiate in Dental Surgery. Its final edition was published in 1925.^5^ In this, dentists could still draw attention to an affiliation which was denied them by the Dental Board of the UK General Medical Council.

## History of the DDS UPenn

The first United States (USA) dental degree was established by the Baltimore Dental College in 1840, but this was not associated with any university; that distinction goes to the DMD Harvard established in 1867.

In 1877, the Medical Faculty of UPenn proposed that a department of dental medicine be established. In the following year, the two pre-existing Pennsylvanian dental schools - the Pennsylvania College of Dental Surgery and the Philadelphia College of Dentistry - agreed to merge with the proposed Department of Dentistry of UPenn, which came into being in April of that year. In total, 25 out of the first entry of 52 students gained their DDS UPenn in 1879. Many of them had transferred from the Pennsylvania College of Dental Surgery but the successful graduates also included two from France and one each from Germany, Switzerland, Italy, and William J. O'Daherty who was a resident of Dublin, then still part of the UK (the Anglo-Irish Treaty of 1922 led to the Irish Free State [now the Republic of Ireland] seceding from the UK).^6^ Perhaps because the latter held no registerable dental qualification in the UK, his name does not appear on any UK dental register or directory. Between 1880-1885, seven successful DDS UPenn graduates are recorded with addresses in the UK; although, two had been born in Philadelphia.

Initially, the Pennsylvania undergraduate dental course was of two years duration. Successful applicants had to be of at least 21 years of age and have previously ‘studied under a respectable private practitioner or medical school'. In 1891/2, the course was increased to three years to conform with the revised requirements of the National Association of Dental Faculties of America (NADFA).^7^ In 1917, the course was increased further to four years. However, the same 1891/2 NADFA meeting adopted a proposal from Dr T. W. Brophy of the Chicago College of Dental Surgery that graduates of a recognised dental college would henceforth only be required to complete the final year of the course and most UK entrants subsequently took this route (Table 1).

**Table 1:** The dental qualifications held by UK dentists at the time of achieving their DDS UPenn 1879-1925 (n = 90)

**No qualification**	8				
**LDSRCS Eng**	44	**LDSRCS Edin**	15	**LDS RCPS Glas**	9
**LDSRCS Ire**	3	**LDS Lpool**	5	**BDS Lpool**	4
**LDS Vict**	1	**LDS NZ**	1		

The number of UK dentists benefiting from the DDS UPenn course is likely to have been much greater than the 90 successful graduates shown in Table 1 as, when it was established, the pass rate was only just over 50%. Sadly, no further information has been found on the subsequent careers of such failed DDS individuals.

## British DDS UPenn graduates

It is not clear why the DDS UPenn degree was so popular, nor how students from outside the USA learned of the new course, but by 1881, the influence of American dentistry was already being felt in the British Isles. The Journal of the British Dental Association (the precursor of the British Dental Journal) was, by this time, carrying advertisements for the Dental Cosmos and the American Dental Depot located in Broad Street, London,^8^ as well as for the monthly Pennsylvania Journal of Dental Science, first published in 1874.

A small number of key individuals had a significant influence in encouraging British applicants to apply for admission to the Pennsylvania course. Matthew H. Cryer (1840-1921) had been born in Southport, Manchester, but his family emigrated to America when he was 11 years old. By 1897, he had become the first Chairman of the University of Pennsylvania, Department of Oral Surgery. He is credited with having established the first hospital dental department. Cryer is still remembered in the UK today for his ‘dental elevators'.^9^ In 1903, he returned to England to visit relatives. While there, he by chance met Herbert Highton, a Liverpool dental student, which led to a number of Liverpool graduates undertaking the DDS UPenn course.^10^

Another likely influence was Theodore Victor Smith (1877-1931), a US citizen who had obtained his DDS UPenn in 1899 and was, for a short time, a Demonstrator at the Liverpool School, before moving to London in 1909, where he took the LDSRCS Eng. He then joined the British Dental Association (BDA) and the Odontological Section of the Royal Society of Medicine, and became dentist to the Prince of Wales. He also founded the British Isles University of Pennsylvania Dental Alumni Society.^5^ Similar influences were later brought to bear in Scotland by Charles Kemball (DDS UPenn, 1913), who became Lecturer in Anatomy and Orthodontics in Edinburgh,^11^ and Andrew Wilson, who returned to Glasgow with his DDS in 1914 and after war service was appointed as Lecturer in Orthodontics at the Glasgow Dental School in 1918.^12^ In 1928, he published the first UK undergraduate orthodontic textbook.^13^ This is clear from the preface, but the book seems to have only been used in Scotland.

## Contribution of British DDS UPenn graduates to British dentistry

It would seem that the DDS UPenn was highly regarded in the UK. Of the 90 UK dentists who achieved this degree and then practised in the UK, 27 were appointed as honorary hospital dental surgeons (Table 2) and 15 held teaching appointments in British dental schools (Table 3).

**Table 2:** Honorary dental surgeon appointments held by British UPenn dental graduates as recorded in the Dentists Directory (assistant dental surgeons have not been included)

**Name**	**Date of DDS**	**Appointment**
Newton	1880	Acton Hospital
Fothergill A	1882	Dental Hospital Newcastle upon Tyne
Gibbons JF	1885	Brighton Hospital
Lankester FJ	1890	Waifs and Strays Hospital Leicester
Dunlop J	1894	Kilmarnock Infirmary
Campbell DR	1895	Edinburgh Dental Hospital
Winder H	1900	Dental Hospital Ireland
Pearce FJ	1901	Guy's Hospital; Belgrave Children's Hospital
Pollitt GP	1901	Military Hospital London; Children's Hospital Paddington
Dorrell HL	1902	Kitchener Indian Hospital
Mellersh F	1904	Royal Dental Hospital
Chapman H	1905	The London Hospital
Armitage JJ	1907	Brighton and Hove Hospital
Porteous	1907	London Hospital
Dagger H	1908	Newton Abbott Hospital
Elliott SG	1908	Indian Hospital Royal Pavillion Brighton
Cooper SS	1908	Bradford Children's Hospital
Crockett LM	1910	Northants Mental Hospital
Husband AP	1912	Glasgow Dental Hospital
McPhee G	1912	Deeside Hydropathic Hospital
Slade RA	1914	Essex County Hospital, Colchester
Crane WA	1915	Royal Victoria Hospital Bournemouth
McMahon	1918	Beckenham Cottage Hospital
Fitton A	1920	Carshalton, Beddington and Wallington Hospitals
Downie GG	1921	Bootle Hospital
Cullen	1921	Royal Dental Hospital
Wallis FR	1922	Margate Hospital

**Table 3:** Teaching appointments held by British DDS graduates

**Name**	**Date of DDS**	**Appointment**
Campbell DR	1895	Lecturer, Edinburgh Dental School
Headridge JP	1896	Lecturer, Manchester Dental School
Pearce FJ	1901	Lecturer, Guy's Hospital Dental School.
Visick HC	1903	Demonstrator, Guy's Hospital Dental School
Chapman H	1905	Lecturer, London Hospital Dental School
Elliot S	1908	Demonstrator, Guy's Hospital Dental School
Pollitt GP	1911	Lecturer, Guy's Hospital Dental School
Hoyes GT	1911	Demonstrator, London Hospital Dental School
Elwood H	1912	Lecturer, Queen's University Belfast Dental School
Husband AP	1912	Lecturer, Glasgow Dental School
Kemball CH	1913	Lecturer in Anatomy and Orthodontics, Edinburgh Dental School
Wilson AG	1914	Lecturer, Glasgow Dental School
Baldwin JA	1920	Demonstrator Liverpool Dental School
Fenn NR	1920	Professor of Dental Prosthetics, Liverpool and later, Guy's Dental School
Child CH	1920	Clinical Lecturer, Leeds Dental School

The General Alumni Catalogue of the University of Pennsylvania was not published after 1922 and the UK Dental Surgeons Directory ceased publication in 1925. Hence, the number and influence of British dentists achieving the DDS UPenn after this date cannot be ascertained. One reason suggested for the discontinuation of the Alumni Catalogue was that the large graduating classes were running into thousands by that time.^14^

**Fig. 1:**
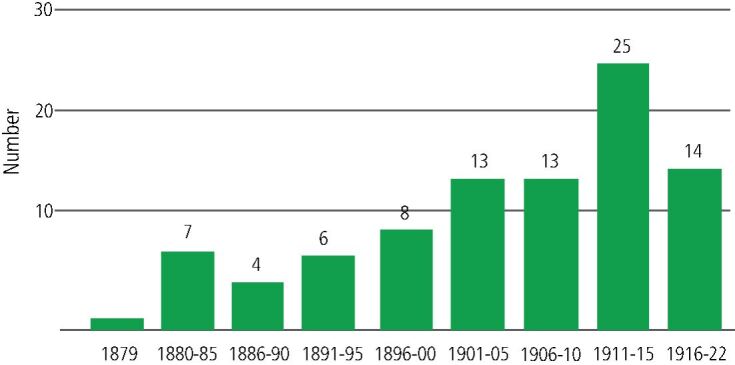
The number of dentists recorded in the general alumni catalogue of the University of Pennsylvania of 1922 as having a UK address, who achieved the DDS UPenn 1880-1922

Of the original 110 dentists recorded in the General Alumni Catalogue of the University of Pennsylvania in 1922 as having a UK address and who achieved the DDS UPenn between 1880-1922, two died in the First World War, one retired from practice due to ill health, 15 emigrated, mostly to Australia and New Zealand, and two practised in the UK for a few years. This left a sample size of 90 (Fig. 1).

The need for postgraduate dental education in the UK did not begin to become acknowledged by the Dental Board until after the Second World War.^15^ By this time, there was already the non-specialist Higher Dental Diploma offered by the Royal College of Surgeons of Edinburgh (1919) and Royal College of Physicians and Surgeons of Glasgow (1920), both of which had been registrable as an additional dental qualification in the UK Dentists Register. However, specialist dental education and training began in America long before that.

## Specialist postgraduate dental programmes of UPenn

### Oral surgery

The $12M legacy of Thomas Wiltberger Evans in 1897 enabled the Pennsylvania School of Dental Medicine to be greatly enlarged.^16^ New staff were appointed, including James G. Lane as Assistant Professor of Orthodontics and Matthew H. Cryer as the first Professor of Oral Surgery. In 1914, Cryer was elected an honorary member of the BDA and in 1916, he established a one-year UPenn postgraduate training programme for those dentists seeking to become oral surgeons. In the following year, the Cryer Society of Oral Surgery was set up.^17^ Several of the British holders of the DDS UPenn were later to become members of that Society.

### Orthodontics

Edward Angle, the widely acknowledged ‘father of orthodontics', had graduated from Pennsylvania College of Dental Surgery in 1878. He subsequently greatly influenced John Mershon, who studied under him in 1908, and who later became the first Chairman of University of Pennsylvania's Orthodontic Department (1916-1924). There was no postgraduate orthodontic course in America at this time, apart from that run by Edward Angle himself,^18^ but under Mershon's influence, there was a significant orthodontic component included in the DDS UPenn program. Ten years later, in October 1925, Mershon presented a paper to the British Society of the Study of Orthodontics.^19^ In the discussion which followed, he described how UPenn now had a one-year orthodontic postgraduate course which had recently been set up by his successor, A. LeRoy Johnson. He reported that the first students had been admitted to the new course in September 1924, which he believed was the only place where currently ‘men could be educated as well as trained in the specialty'. The course content is described in the paper by Grey.^20^ On successful completion of the course, these postgraduate students only received a certificate rather than a further university diploma or degree, which makes it very difficult to determine just how many people took what was the first specialist orthodontic course run by a university. It is clear that British dentists, such as Brigadier Graeme Matthew Warrick CBE, attended this course until the Second World War.^21^

The British Society for the Study of Orthodontics (BSSO) which, in 1994, merged with other orthodontic societies to become the British Orthodontic Society, was founded in 1907 by George Northcroft. He achieved the DDS Ann Arbor Michigan in 1890. However, the majority of the Society's first key officers were DDS UPenn graduates (Table 4). Their influence may, in part, have contributed to the significant increase in those taking the DDS UPenn in the years between the establishment of the BSSO and the outbreak of the First World War (Fig. 1).

**Table 4:** Early principal officers of the British Society for the Study of Orthodontics

**Role**	**Name**
President 1908	JH Badcock
President 1909	H Chapman DDS UPenn 1905
President 1910	S Wallace
President 1911	W Rushton
President 1912	H Baldwin
President 1913	M Hopson
President 1914	NG Bennett
President 1915	WF Mellersh DDS UPenn 1904
Vice President 1908	G Northcroft DDS Ann Arbour
Secretary (1908-1910)	AC Lockett DDS UPenn 1905
Secretary (1911-1924)	H Chapman DDS UPenn 1905
Treasurer (1908-1913)	WF Mellersh DDS UPenn 1904
Treasurer (1914-1922)	HG Highton DDS UPenn 1906
Librarian (1909-1912)	JE Spiller
Curator (1908-1910)	H Visick DDS UPenn 1903

## Other contributions of UPenn to British dentistry

In total, 16 of the 90 DDS UPenn graduates who returned to practise in the UK had published papers in major journals by 1925, and four went on to produce books in their practising lifetimes. Six became Presidents of their local BDA branch, and many more became officers of local dental societies and study groups. Four were made life members of the BDA and three rose to become the BDA National President: F. J. Pearce (1936), H. Elwood (1938) and A. P. Husband (1955). Finally, one became President of the Royal Society of Medicine (H. Chapman, 1944).

## Conclusion

While it is impossible to separate the effect of attending the DDS UPenn course from the character and efforts of individuals themselves, there is little doubt that graduates from UPenn had more influence on UK dentistry than from any other American dental school.

## References

[1] Gelbier S. 125 years of developments in dentistry, 1880-2005. Part 5: dental education, training and qualifications. *Br Dent J* 2005; **199:** 685-689. 10.1038/sj.bdj.4813002 16311582

[2] Gelbier S. Dentists Registers: 1879-1925. *Br Dent J* 2022; **232:** 55-58. 10.1038/s41415-021-3820-3 35031747

[3] Asbell M B. *A century of dentistry - a history of the University of Pennsylvania School of Dental Surgery.* Philadelphia: University of Pennsylvania, 1977.

[4] Dental Board of the United Kingdom. *Names and addresses of dental practitioners registered in the United Kingdom - foreign list*. London: Dental Board of the United Kingdom, 1925.

[5] Anonymous. *The dental surgeons directory 1925.* London: J. A. Churchill, 1925.

[6] UK Parliament. The Anglo-Irish Treaty 1921. 2022. Available at https://commonslibrary.parliament.uk/research-briefings/cbp-9260/ (accessed February 2025).

[7] National Association of Dental Faculties. Report of the 30^th^ annual meeting of the National Association of Dental Faculties. *Am J Dent Sci* 1896*;* **30:** 274*-*283. PMC6118455 30757538

[8] Journal of the British Dental Association. Advertisement. *J Brit Dent Assoc* 1881; **2:** ii.

[9] Bussell M A, Graham R M. The history of commonly used dental elevators. *Br Dent J* 2008; **205:** 505-508. 10.1038/sj.bdj.2008.933 18997710

[10] Chapman H. Fifty years in retrospect. *Transact Br Soc Study Orthod* 1954; **40:** 100-116.

[11] British Dental Journal. Obituary. *Br Dent J* 1964*;* **116:** 456.

[12] Henderson T B. *The history of the Glasgow Dental Hospital and School 1879-1979*. Glasgow: Glasgow Dental School, 1980.

[13] Wilson A G. *The outlines of dental science: orthodontics.* Vol 12. Edinburgh: E & S Livingstone, 1928.

[14] Setton K M (UPenn Librarian). Letter to Brigham C D (Director, American Antiquarian Society). 12 February 1958.

[15] British Dental Journal. Announcement: post-graduate education. *Br Dent J* 1948; **84:** 221.

[16] Hughes S. Crowns and confidences. 1999. Available at https://thepenngazette.com/crowns-and-confidences/ (accessed November 2024).

[17] Hendler B H, Quinn P D. University of Pennsylvania oral and maxillofacial surgery training program. *J Oral Maxillofac Surg* 2009; **69:** 937-942. 10.1016/j.joms.2007.03.035 19382388

[18] Peck S. The students of Edward Hartley Angle, the first specialist orthodontist: a definitive compilation. *J Hist Dent* 2006; **54:** 70-76. 17039863

[19] Mershon J V. A practical talk on why the lingual arch is applicable to the orthodontic problem. *Transact Br Soc Study Orthod* 1925; 58-63.

[20] Grey N. My work and experiences at the postgraduate school of orthodontics University of Pennsylvania. *Transact Br Soc Study Orthod* 1926; 125-136.

[21] University of Pennsylvania. *Dental medicine catalogue 1937/8.* Philadelphia: University of Pennsylvania, 1938.

